# Integrated Metabolome and Transcriptome Analysis Provides New Insights into the Glossy Graft Cucumber Fruit (*Cucumis sativus* L.)

**DOI:** 10.3390/ijms241512147

**Published:** 2023-07-29

**Authors:** Jie Ren, Lu Yang, Ruifang Cao, Yidan Wang, Can Zhang, Xuejing Yu, Wendi Meng, Xueling Ye

**Affiliations:** College of Horticulture, Shenyang Agricultural University, 120 Dongling Road, Shenhe District, Shenyang 110866, China; 2019500023@syau.edu.cn (J.R.);

**Keywords:** *Cucumis sativus* L., wax powder, graft, differentially expressed genes

## Abstract

Cucumber is an important vegetable crop, and grafts often affect the quality and wax loss in cucumber fruit and affect its value. However, their metabolites and molecular mechanisms of action remain unclear. Metabolome and transcriptome analyses were conducted on the fruit peels of self-rooted plants (SR) grafted with white seed pumpkin (WG). The results showed that there were 352 differential metabolites in the fruit peels of the SR and WG. The transcriptome analysis showed 1371 differentially expressed genes (DEGs) between the WG and SR. These differentially expressed genes were significantly enriched in plant hormone signal transduction, cutin, suberin, wax biosynthesis, phenylpropanoid biosynthesis, and zeatin biosynthesis. By analyzing the correlation between differential metabolites and differentially expressed genes, six candidate genes related to the synthesis of glycitein, kaempferol, and homoeriodictyol were identified as being potentially important. Key transcription factors belonging to the TCP and WRKY families may be the main drivers of transcriptional changes in the peel between the SR and WG. The results of this study have provided a basis for the biosynthesis and regulation of wax loss and quality in grafted cucumbers and represents an important step toward identifying the molecular mechanisms of grafting onto cucumber fruit.

## 1. Introduction

Cucumber (*Cucumis sativus* L.) is an annual herbaceous plant belonging to the Cucurbitaceae family. It has wide diversity, and the best area and yield worldwide. Waxy fruit coverage is one of the defining qualities of high-quality cucumber products [1]. Wax powder is a noncellular structural substance attached to the epidermal tissues of plants. It typically has a hydrophobic layer that acts as a natural protective barrier and is gray-green or gray-white frost-like, comprising several compounds that are soluble in organic solvents but insoluble in water [2]. Wax powder has a crystal structure and is characterized by drought resistance. Wax powder on the surface of cucumbers can reduce the adhesion ability of insects, which affects feeding by insects and protects the cucumber fruit [3]. However, the presence of an excessive layer of wax powder on the cucumber epidermis impairs the quality of the fruit, which lowers its value [4]. As the vegetable industry has grown, cucumber types with little or no wax powder have become more popular among customers. Therefore, it is crucial to examine the wax on the surface of cucumber fruits.

Given the importance of wax powders, many researchers have studied the formation mechanism of cucumber surface waxes. Cucumber fruit wax powder is a layer of a white powdery substance that adheres to the surface of the cucumber fruit and comprises four to eight seedless cells with a silicified spherical surface [5]. Wax powder is originally divided into hair cells from the vertical surface of a single cell of the pre-anthesis young fruit peel and then divided into stalks and grains. With the enlargement of the post-anthesis fruit, the grain cells divide into globules composed of 6–8 cells filled with inclusions, and the nuclei gradually disappear. When the fruit reaches commercial maturity, a layer of inclusions precipitates on the surface of the globular body and is dispersed on the surface of the fruit with the globular body to form a white wax powder. When the fruit has fully matured, the wax powder gradually falls off. However, the surface of wax powder globular bodies of cucumber varieties with more wax powder is covered with a layer of silicon, and not all cucumber wax powder globular bodies precipitate inclusions. The surface of wax powder globular bodies in cucumber varieties with less wax powder has no silicon. When the fruit expands and matures, the wax powder of the cucumber fruit surface with less wax powder gradually decreases. This then indicates that the fruit has reached maturity [6]. To date, the composition of wax powder has mainly been determined using gas chromatography–mass spectrometry (GC-MS). Mass spectrometry–gas chromatography analysis has shown that the main components of cucumber peel wax are lipids and alkanes containing small amounts of phenols, alcohols, and olefins. Lipids are mainly phthalates and alkanes are mainly C12–C33 compounds [7]. Pan et al. (2018) detected 201 compounds in cucumber peels using metabolomic analysis, including 16 flavonoids and 14 phosphate esters.

Several factors affect the formation of cucumber epidermal wax powder, including genetic factors, temperature, humidity, light, and the pumpkin rootstock. The presence or absence of wax powder in cucumber is controlled by a pair of major genes, and the presence or absence of wax powder is either dominant or partially dominant. In addition to the main gene controls, there are other related modification genes. Wax powder is closely related to temperature. The lower the air temperature, the less wax powder is present on the fruit surface [8]. Humidity interacts with temperature to influence the formation of wax powder. At higher temperatures, the amount of wax powder decreases with increasing humidity [9]. Meanwhile, at lower temperatures, the amount of wax powder increases with increasing humidity [10]. However, with fruit growth, the wax powder on the surface of the cucumber fruit grafted with the rootstock “Huofenghuang” gradually decreases or disappears, and the peel color is relatively bright. The Yunnan black seed pumpkin, Qingyan Rootstock No. 1, grafted on cucumbers results in a wax powder with a dim color on the fruit surface. This indicates that wax powder is closely related to cucumber varieties and grafted rootstocks. After cucumber varieties with more wax powder are grafted onto appropriate rootstocks, the amount of wax powder is substantially reduced. The ability to remove wax powder from pumpkins with black seeds is less pronounced. Although grafting can affect the wax powder content in the cucumber epidermis, the metabolic pathways and regulatory sites that change following grafting have not yet been determined.

Therefore, in this study, white-seeded pumpkin with multi-wax powder rootstock was grafted with cucumber scion (“Zhongnong 18”) with high wax powder content to examine the effect of white-seeded pumpkin rootstock grafting on wax powder on cucumber fruit surfaces. From the perspectives of transcriptomics and metabolomics, the potential internal mechanism of white-seeded pumpkin rootstock grafting leading to an increase in wax powder on the cucumber scion fruit surface was analyzed.

## 2. Results

### 2.1. Phenotype of WG and SR Cucumber Fruits

**Figure 1:**
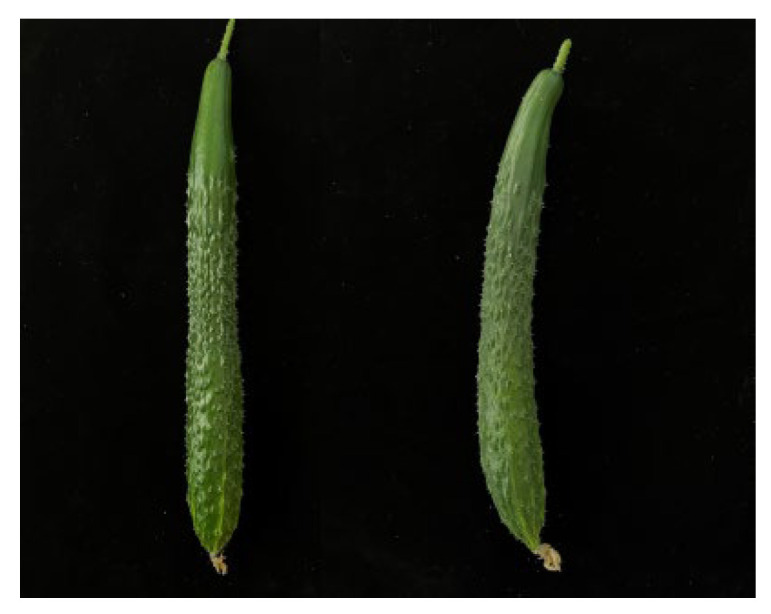
Fruit phenotype of wax powder on the fruit surface of WG and SR. The phenotype of fruit in WG (**left**) and SR (**right**) at 12 d after flowering.

Commercially mature self-rooted (SR) cucumber fruits have thick wax powder coatings. The amount of wax powder on the surface of white seed pumpkin-grafted cucumber (WG) was lower than that on matching self-rooted cucumbers. The fruit surface was clear and bright, and the skin color of the fruit could be clearly identified (Figure 1).

The differences in brightness between the SR and WG were evaluated at 3, 6, 9, 12, and 15 d after flowering. As shown in Table 1, the wax powder content in the SR epidermis steadily increased with plant growth and development. Twelve days after flowering, the brightness difference in the fruit surface reached 1.35, and the wax powder content decreased. However, the WG fruit surface brightness difference peaked six days after blooming and then steadily decreased. We examined the variation in wax powder brightness on the fruit surfaces of the WG and SR plants at various phases (Table 1). The results showed that the WG and SR fruit surfaces had different wax powder brightness levels. The findings demonstrated that at 9, 12, and 15 d after flowering, there was a substantial difference in the brightness of the wax powder on the fruit surfaces of the WG and SR, with the difference reaching its peak on Day 12 following flowering. It is important to examine how grafting affects the production of wax powder on fruit surfaces.

**Figure 2:**
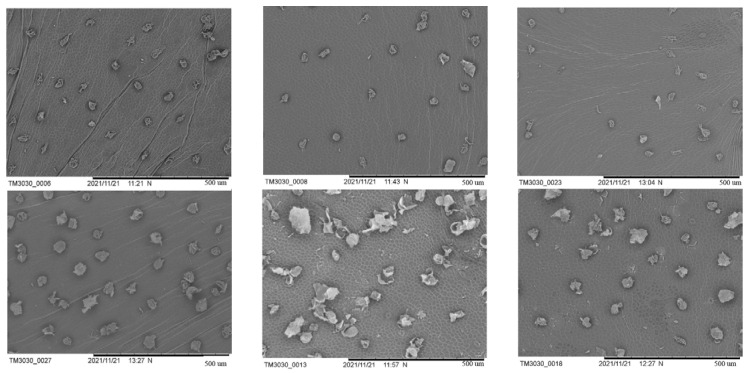
SEM analysis of WG and SR at 9, 12, and 15 d after flowering.

Cucumber wax powder was observed using scanning electron microscopy (SEM) during the period when its shape and structure underwent significant alterations (9, 12, and 15 d after flowering) (Figure 2). On the ninth day after flowering, the SR had wax powder at various stages of development, some of which were young and still forming, and the density of the wax powder was relatively high. Although there was a lower amount of freshly created wax powder on the fruit surface of the WG than that of the SR and lower density, the differences were not significant. At the WG growth stage, the SR had more wax powder, while the mature wax powder of the WG had a higher degree of depression, a rougher surface, and a clear silicified layer. Both also contained sticky residues from wax powder particles that split apart with expansion of the peel. SR wax powder was still present on the 12th day after flowering at various stages of development. However, it was more mature and had a lower density than it had on the 9th day. There were still several phases of wax powder generation in the WG, and there were few freshly created tiny wax powders. The density of the wax powder in all the cucumbers was lower than it had been nine days after flowering because of the separation of the wax powders from each other. At 9 and 12 d after flowering, the wax powder depression was less severe than that in the SR. The silicified layer of the wax powder globules cracked on the surface of the fully formed and mature globular cells. During the development stage, no visible breaking of the wax powder was observed. The exocarp cell outline, which was spotless and new, was clearly visible on the fruit surface. The pericarps of the SR and WG included pieces and cracks, and in both cases, sticky traces of wax powder particles separated as the pericarp extended. On the day following flowering, a few wax granules developed in the SR, and their sizes varied. Compared with the 12th day after flowering, the wax powder density was sparser, and the amount of relatively mature large wax particles increased. Compared to the 12th day after flowering, the number of fractured fragments and pericarp fragments significantly decreased. The wax powder of the grafted cucumber did not significantly increase in density compared to 12 d after flowering, and the silicified layer of the wax powder was much lighter than the SR from the earlier stages and in the same period. The fruit surface was clean and fresh, and the outline of the exocarp cells could be clearly seen.

In summary, the WG wax powder with strong dewaxing power has a smooth surface, neat structure, thin silicified layer, small individual cucumber wax powder particles, and low density. Combined with scanning electron microscopy and surface brightness difference values, 12 d post flowering samples were selected for metabolome and transcriptome analysis.

### 2.2. Differential Accumulation of Metabolites between SR and WG

**Figure 3:**
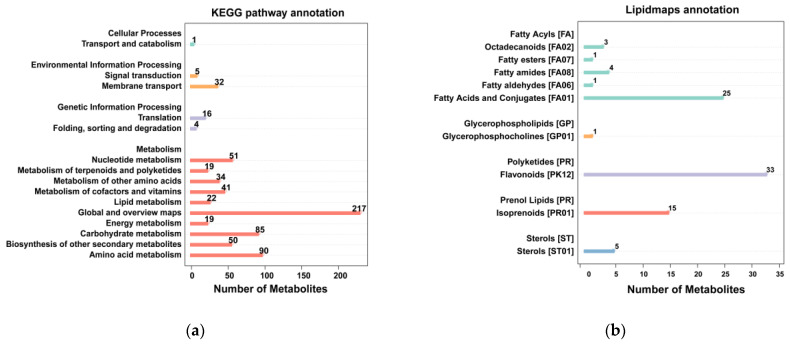
KEGG and LIPID MAPS analysis in WG vs. SR. (**a**) KEGG analysis in WG vs. SR; (**b**) LIPID MAPS analysis in WG vs. SR.

**Figure 4:**
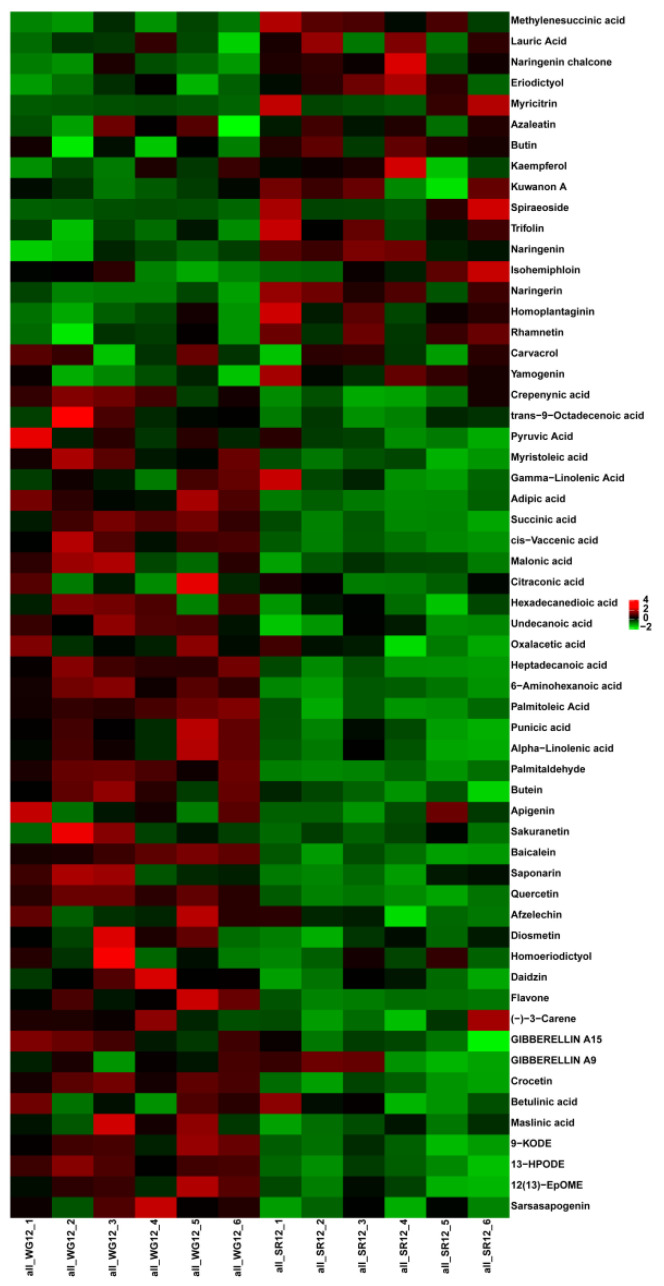
Differentially accumulated metabolites in WG vs. SR. Each row represents a metabolite, and each column represents a biological repeat of WG or SR. The closer the color of the square to red, the higher the expression level of metabolites. On the contrary, the closer the color of the square to green, the lower the expression level of metabolites. The heatmap was generated using online website (https://www.omicshare.com/ (accessed on 19 May 2023)).

In total, 1014 metabolites were detected in the WG and SR using the LC-MS analysis platform. The metabolites identified were annotated by using the main databases, including KEGG (Kyoto Encyclopedia of Genes and Genomes) and LIPID MAPS, to understand the functional characteristics and classification of different metabolites. According to the KEGG enrichment results, 686 metabolites were aligned with lipid metabolism and the biosynthesis of other secondary metabolites (Figure 3a). A total of 34 metabolites were enriched in the lipid maps, including 34 fatty acyls, one glycerophospholipid, 33 polyketides, 15 prenol lipids, and five sterols (Figure 3b). The PCA (principal component analysis) analysis and PLS-DA (partial least squares–discriminate analysis) were used to identify the differentially accumulated metabolite (DAM), and 352 differentially accumulated metabolite were identified. Among them, 258 were upregulated and 94 were downregulated. We found that one DAM (sn-Glycero-3-phosphocholine), one DAM (Palmitoleic Acid), and three DAMs (9(S)-HOTrE, 13(S)-HOTrE, and 9(S)-HpOTrE) were involved in ether lipid metabolism, fatty acid biosynthesis, and alpha-linolenic acid metabolism, respectively (Figure 4).

### 2.3. Identification of Differentially Expressed Genes

To further elucidate the molecular basis of differential wax accumulation in fruits, we conducted a comparative transcriptome analysis using Illumina sequencing technology. Six libraries were created and sequenced from three biological replicates for each SR and WG group six days after flowering. After quality control and filtering for each sample, we yielded clean base values of 6.46 G to 7.01 G, Q20 values of 96.83–97.63%, and Q30 values of 91.7–93.37%, with a GC pct range of 44.19–45.08 (Table S1). Unique map reads varied from 91.41% to 92.77%, whereas total map reads fell between 94.61% and 95.85%. The multimap readings were lower than 10%, ranging from 2.99% to 3.38% (Table S2). These findings indicated that the sequencing data were accurate and could be applied to the study of gene expression.

**Figure 5:**
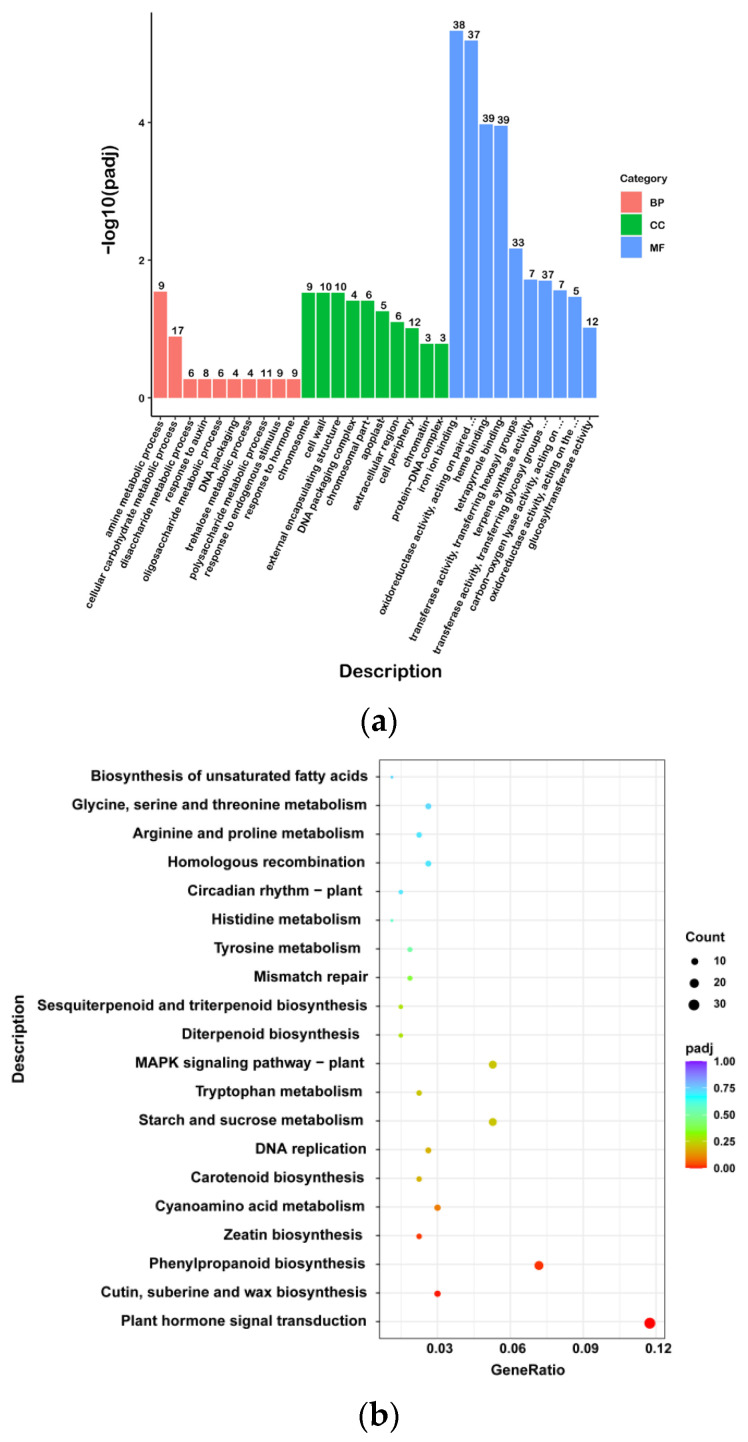

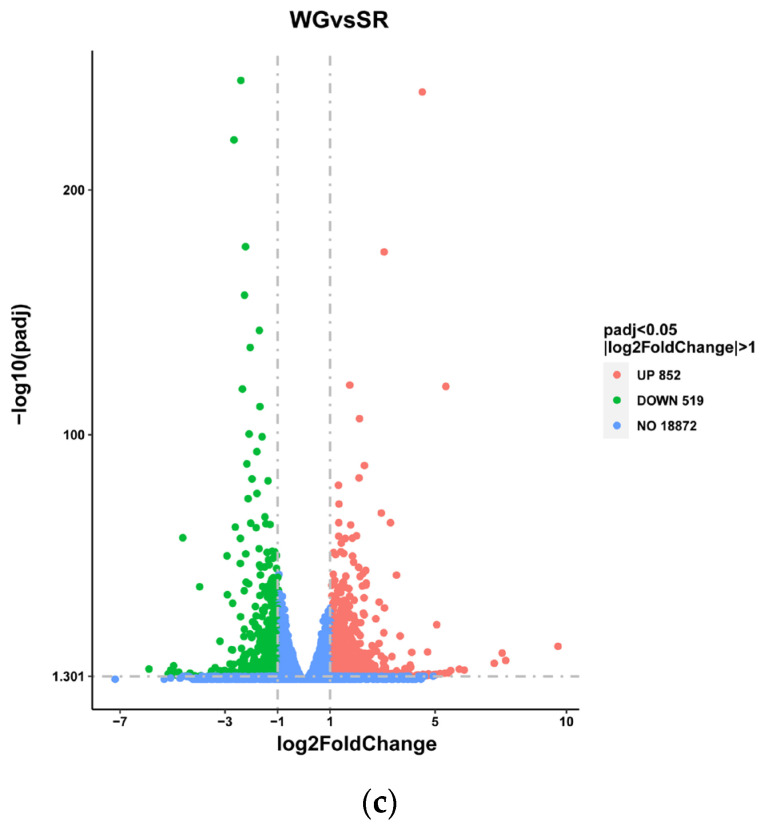
RNA-seq analysis in WG vs. SR. (**a**) Differentially expressed gene volcano map; (**b**) GO enrichment analysis histogram; (**c**) KEGG enrichment scatter diagram.

We calculated the expression values (FPKM) for all the genes in each sample according to the reads. The significantly differentially expressed genes were identified according to the threshold of FDR ≤ 0.05 and |log2Ratio| ≥ 1. In WG versus SR, there were 1371 DEGs (852) and downregulated (519) (Figure 5a), indicating that these DEGs may be involved in wax accumulation in fruits.

### 2.4. Functional Classification of the DEGs by Enrichment Analysis

The Gene Ontology (GO) enrichment analysis of the DEGs was performed and significantly enriched GO terms in the differentially expressed genes were screened (Figure 5b). The DEGs were enriched in 590 GO terms and were significantly enriched in 15 GO terms. In the biological process category, the largest class was the “amine metabolic process”, and nine DEGs were enriched in the WG and SR. Among the molecular function categories, 39, 39, and 38 DEGs were enriched for “heme binding”, “tetrapyrrole binding”, and “iron ion binding”, respectively. In the cellular component category, 10 and 10 DEGs in WG vs. SR were enriched in the “cell wall” and “external encapsulating structure”, which was the largest class. All the DEGs were significantly enriched in 15 GO terms, 1 biological process term, 5 cellular component terms, and 9 molecular function terms, including “amine metabolic process”, “chromosome”, “cell wall”, “external encapsulating structure”, “DNA packaging complex”, “chromosomal part”, “iron ion binding”, “oxidoreductase activity, acting on paired donors, with incorporation or reduction of molecular oxygen”, “heme binding”, “tetrapyrrole binding”, “transferase activity, transferring hexosyl groups”, “terpene synthase activity”, “transferase activity, transferring glycosyl groups”, “carbon-oxygen lyase activity, acting on phosphates”, and “oxidoreductase activity, acting on the CH-NH group of donors”.

The upregulated DEGs were categorized into 522 GO terms. In the biological process category, the largest class was the carbohydrate metabolic process, and 27 DEGs were enriched in the WG and SR. Among the molecular function categories, 22 DEGs were enriched in “hydrolase activity”, which acts on ester bonds. In the cellular component category, 16 DEGs in WG versus SR were enriched in the nucleus, which was the largest class. All DEGs were significantly enriched in 14 GO terms, 10 cellular component terms, and four molecular functions, including “chromosome”, “cell wall”, “external encapsulating structure”, “chromosomal part”, “DNA packaging complex”, “cell periphery”, “extracellular region”, “apoplast”, “chromatin”, “protein-DNA complex”, “terpene synthase activity”, “carbon-oxygen lyase activity, acting on phosphates”, “iron ion binding”, and “oxidoreductase activity, acting on paired donors, with incorporation or reduction of molecular oxygen”. Cutin, suberin, and wax biosynthesis were enriched in eight DEGs in WG versus SR, including five upregulated and three downregulated DEGs.

The downregulated DEGs were categorized into 324 GO terms. In the biological process category, the largest class was transmembrane transport, and 15 DEGs were enriched in the WG and SR. Among the molecular function categories, 22 DEGs were enriched in transcriptional regulator activity. In the cellular component category, eight DEGs in WG vs. SR were enriched in the nucleus, which was the largest class. All DEGs were significantly enriched in eight molecular function terms, including “oxidoreductase activity, acting on paired donors, with incorporation or reduction of molecular oxygen”, “iron ion binding”, “heme binding”, “transferase activity, transferring hexosyl groups”, “tetrapyrrole binding”, “transferase activity, transferring glycosyl groups”, “extracellular region”, “DNA-binding transcription factor activity”, and “transcription regulator activity”.

A Kyoto Encyclopedia of Genes and Genomes (KEGG) enrichment analysis was performed to further understand the molecular interactions of DEGs between the WG and SR (Figure 5c). The DEGs were enriched in 96 pathways and significantly enriched in 4 pathways, which were “Plant hormone signal transduction”, “Cutin, suberine and wax biosynthesis”, “Phenylpropanoid biosynthesis”, and “Zeatin biosynthesis”. For the upregulated DEGs, the DEGs in WG vs. SR were enriched in 86 pathways, and there were no significantly enriched pathways. For downregulated DEGs, the DEGs in WG vs. SR were enriched in 56 pathways and significantly enriched in 3 pathways, which were “Plant hormone signal transduction”, “Phenylpropanoid biosynthesis”, and “Zeatin biosynthesis”.

### 2.5. Differentially Expressed Genes Involved in Wax Accumulation

**Figure 6:**
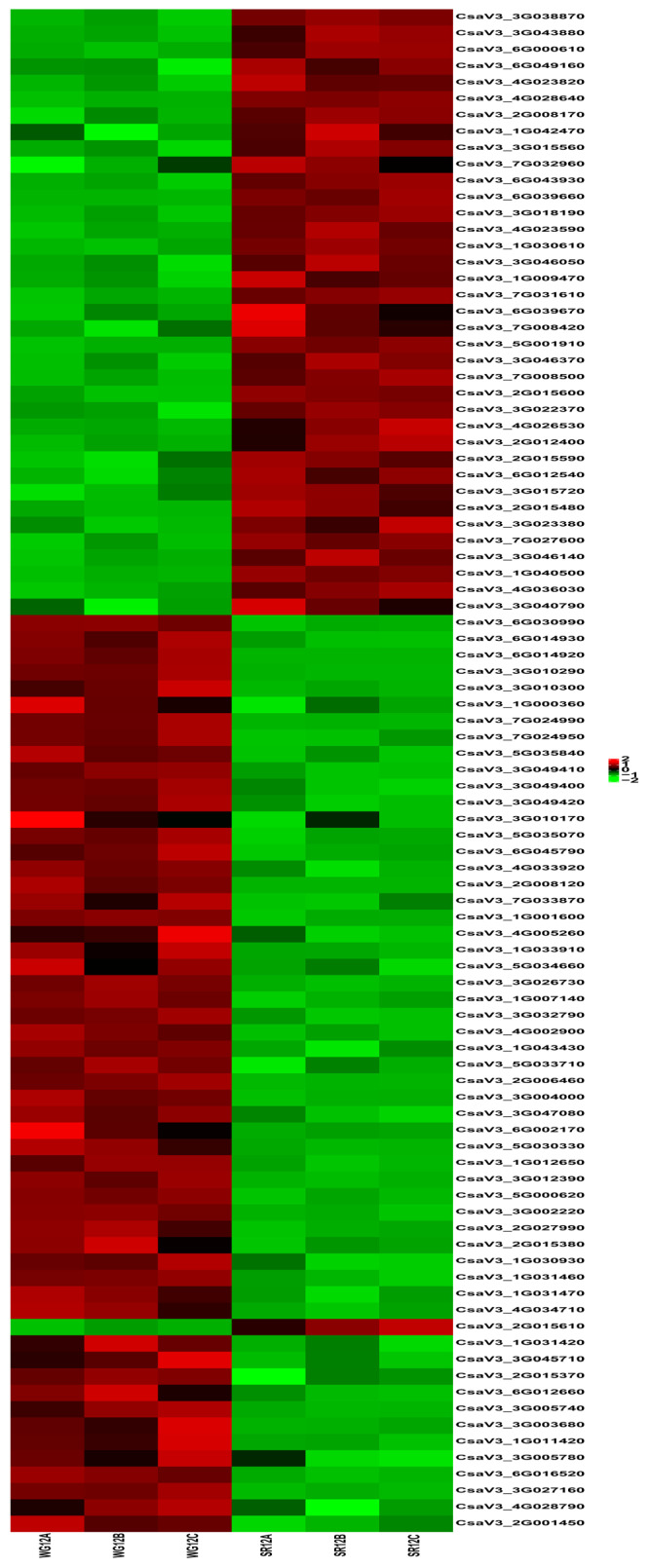
Expression results of differentially related genes involved in wax synthesis. Each row represents a gene, and each column represents a biological repeat of WG or SR. The closer the color of the square to red, the higher the expression level of metabolites. On the contrary, the closer the color of the square to green, the lower the expression level of metabolites.

To identify the key genes involved in cuticular wax loss in the WG, the gene expression patterns of the DEGs were analyzed based on their functions. For the enrichment analysis, we selected 41 DEGs involved in fatty acid metabolism and lipid transport and metabolism pathways, such as the fatty acid degradation, a-linolenic acid metabolism, diterpenoid biosynthesis, sesquiterpenoid and triterpenoid biosynthesis, biosynthesis of unsaturated fatty acids, and so on (Table 2, Figure 6). Among these, 33 were upregulated and 7 were downregulated. The 33 upregulated DEGs were mainly involved in all the pathways mentioned above. Meanwhile, the eight DEGs were predominantly involved in cutin, suberin, and wax biosynthesis, fatty acid metabolism, a-linolenic acid metabolism, and Glycerolipid metabolism, including fatty acid omega-hydroxy dehydrogenase, CYP450, fatty acid omega-hydroxylase, diacylglycerol O-acyltransferase 3, D-glycerate 3-kinase, and lipoxygenase; cutin, suberin, and wax biosynthesis were enriched in eight DEGs in WG versus SR, including five upregulated and three downregulated DEGs.

**Figure 7:**
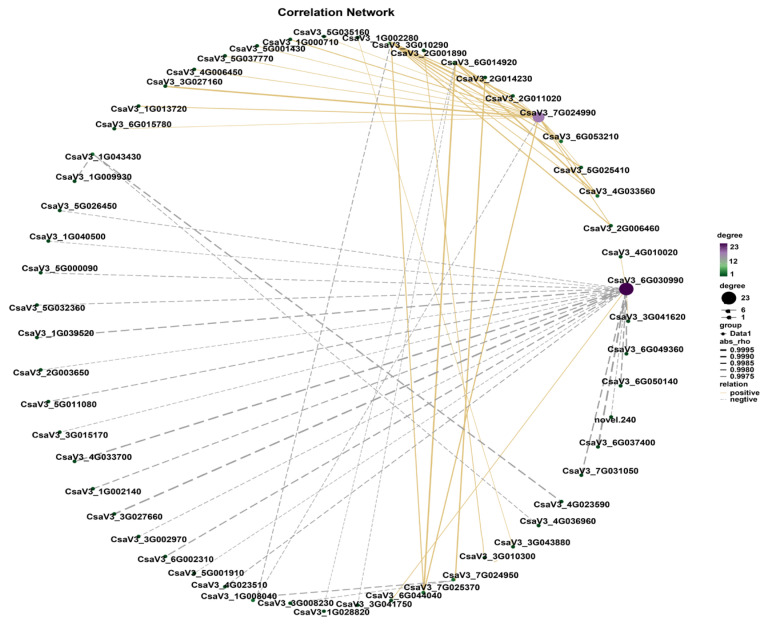
The co-expression network of DEGs involved in wax-associated metabolism and transcription factors. The gray arrows indicate a negative correlation, and the yellow arrows indicate a positive correlation. The size of the circles was determined based on the degree of connectivity.

Transcription factors (TFs) regulate wax accumulation. In this study, 536 differentially expressed TFs were identified, of which 303 were upregulated and 233 were downregulated. A co-expression network of the DEGs was built based on their expression patterns. Gene pairs with *p*-values < 1 × 10^−5^ 4004 were considered to show consistent correlations. As shown in Figure 7, there were many interconnections between the TFs and DEGs mentioned above, indicating that wax accumulation is a coordinated process. Among these, *WRKY53* (*CsaV3_7G025370*) was positively correlated with upregulated *fatty acyl-CoA reductase 3* (*CsaV3_6G014920*) and *CER1 (ECERIFERUM1)* (*CsaV3_3G010290*). *CBL-interacting serine/threonine protein kinase 6* (*CsaV3_1G028820*) was negatively correlated with the upregulated *fatty acyl-CoA reductase 3* (*CsaV3_6G014920*). *TCP14* (*TEOSINTE BRANCHED1, CYCLOIDEA, and PCF 14*) (*CsaV3_1G002280*) expression negatively correlated with downregulated *GMC oxidoreductase* (*CsaV3_3G043880*) expression.

### 2.6. qRT-PCR Validation of Differentially Expressed Genes

**Figure 8:**
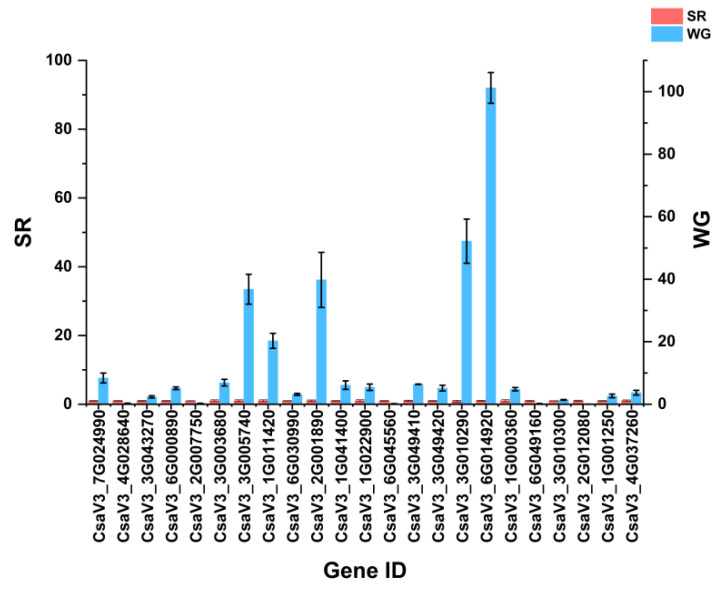
Expression levels of the DEGs related to wax biosynthesis at S0–S2. Data are represented as mean ± standard deviation.

To further confirm the reliability of the transcriptome data, 24 DEGs were selected for qRT-PCR analysis. A total of 24 DEGs related to the fatty acid biosynthetic process, long-chain fatty acid metabolic process, long-chain fatty acid metabolic process, lipid metabolic process, a-linolenic acid metabolism, diterpenoid biosynthesis, sesquiterpenoid and triterpenoid biosynthesis, biosynthesis of unsaturated fatty acids and the ABC transporter family, and three TFs (*WRKY53* (*CsaV3_7G025370*), *TCP14* (*CsaV3_1G002280*), and *CBL-interacting serine/threonine-protein kinase 6* (*CsaV3_1G028820*) were selected for validation (Figure 8). The expression patterns of these DEGs were consistent with the RNA-seq results, further demonstrating the reliability of the sequencing results.

### 2.7. Association Analysis of Genes and Metabolites Related to Wax Deficiency in WG vs. SR

**Figure 9:**
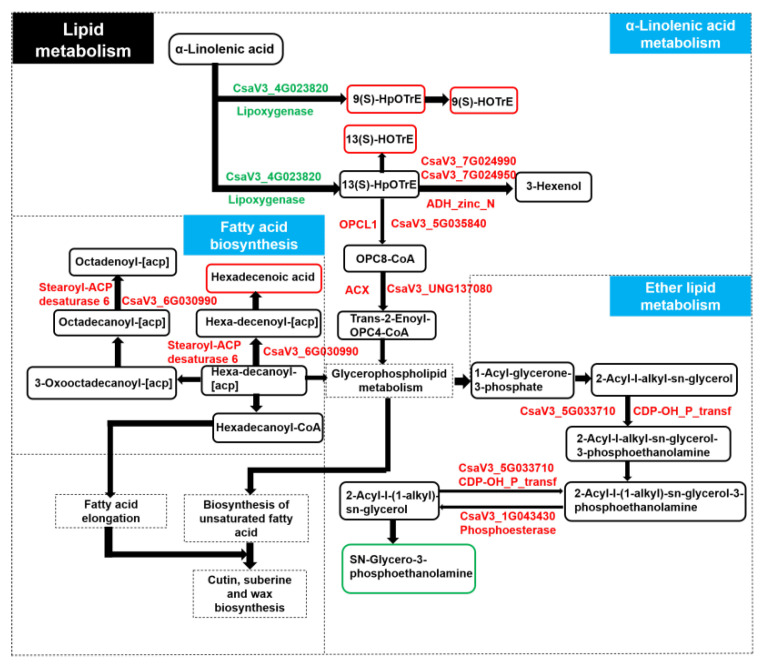
The relationship between genes and metabolites in three pathways.

To better understand the relationship between genes and metabolites, the Pearson correlation coefficient was calculated for the correlation analysis. The DAMs and DEGs in WG vs. SR were then mapped to the corresponding KEGG (http://www.genome.jp/kegg/ (1 January 2023)) pathway to determine the relationship between the key genes and metabolites associated with wax deficiency. We identified one DEG involved in fatty acid biosynthesis, and the upregulated DEG led to an increase in hexadecenoic acid content (Figure 9). The upregulated DEGs involved in ether lipid metabolism led to a decrease in the content of sn-Glycero-3-phosphocholine (Figure 9). Meanwhile, the downregulated DEG (CsaV3_4G023820) involved in a-linolenic acid metabolism led to the increase in the content of 9(S)-HOTrE, 13(S)-HOTrE, and 9(S)-HpOTrE, and further led to the upregulated OPCL, ACX, and alcohol dehydrogenase class-P (Figure 9).

## 3. Discussion

Research on the commodity qualities of cucumber fruit is becoming increasingly crucial, as it is one of the most important fruits in China. The methods of wax production, influencing variables, and content detection have all been disclosed in a growing number of studies. However, most of them have focused on natural mutants and environmental influences. Further investigation into the molecular processes underlying the effects of various pumpkin rootstocks grafted onto wax powder on the surface of cucumber fruits is still needed. Given that WG and SR have different epicuticular wax compositions, we conducted physiological and transcriptomic investigations of the two tissues in this study. The DEGs and DAMs found in the WG versus the SR provided insights into the potential molecular mechanism causing the wax shortage in glossy cucumbers based on a comparison of transcriptome and metabolic data.

In response to environmental obstacles, cuticle wax functions as a chemical and physical barrier. Wax powder coating degrades the commercial quality of cucumbers. No differences have been found in the pericarp wax content between self-rooted cucumbers. However, rootstocks play a substantial role in the development of wax powder on grafted cucumber fruit surfaces. Liu et al. (2014) observed that wax powder content is closely related to cucumber varieties and grafted rootstocks. After the multi-wax powder cucumber varieties were grafted onto appropriate rootstocks, the amount of wax powder was significantly reduced, and the ability of the black seed pumpkin to remove wax powder was less pronounced [11]. Zhu et al. (2018) concluded that black seed pumpkin as a rootstock has a significant increase in the wax powder layer on the surface of the fruit [4]. In this study, we discovered that the self-rooted cucumber had a wax coating, whereas the pericarp of the cucumber grafted onto a white pumpkin had a glossy phenotype (Figure 1).

The wax in cucumbers is ball- or flake-shaped, in contrast to the rod- or stick-shaped waxes in Arabidopsis and other crops [12]. Wax powder was present in the SR at various growth phases and had a high density. Combined with the cuticular wax composition having changed significantly after grafting in this study, we hypothesized that the rootstock affects wax biosynthesis in the scion. However, this hypothesis needs to be validated.

Grafting mechanisms have been studied in several species, including apples, tomatoes, and watermelons [7,11,13,14,15]. The main genes involved in grafted cucumber development have been investigated [16,17]. We analyzed the differentially expressed metabolites in the WG and SR and identified 352 differentially accumulated metabolites. Among them, DAMs involved in ether lipid metabolism, fatty acid biosynthesis, and alpha-linolenic acid metabolism were identified. To deepen our understanding of the relationship between genes and metabolites, the Pearson correlation coefficient was calculated for a correlation analysis. The fatty acid biosynthesis, ether lipid metabolism, and a-linolenic acid metabolism were then discovered. These pathways may also play important roles in wax biosynthesis pathway genes in response to grafting. Research has shown that grafting affects an exchange of microRNA between the scions and rootstocks of grafted watermelon, which may regulate the growth and development of scions [15,18]. There has been extensive research on the metabolites of plant wax powder. Tian et al. (2021) conducted GC-MS detection on red palm bracts and identified a total of 153 differential metabolites related to wax powder, of which 55 were upregulated and 97 were downregulated. Among these differential metabolites, the most metabolites belonged to ketones, followed by organic acids or salts, and the less abundant belonged to alcohols, ethers, phenols, peptides, and amines. These differential metabolites were involved in flavonoid synthesis, fatty acid synthesis, and degradation [19]. The wax components of aromatic pears were also analyzed. A total of 165 differential metabolites were selected, including 106 upregulated metabolites and 50 downregulated differential metabolites. These differential metabolites were mainly enriched in pathways such as fatty acid degradation and terpene quinone biosynthesis, such as ubiquinone (Du, 2019) [20]. In this study, a total of 352 differential metabolites were identified in WG vs. SR. Among them, amino acids and their derivatives were the most abundant among the differential metabolites, with a total of 87 (24.7%), followed by nucleotides and their derivatives, as well as sugars and their derivatives, with 30 (8.5%), flavonoid metabolites with 27 (7.7%), fatty acyl metabolites with 26 (7.4%), and organic acids and their derivatives with 20 (5.7%). Amino acids and organic acids are both hydrocarbon derivatives, and hydrocarbons are one of the important components of wax powder, which are indispensable for the formation of the fruit wax powder structure (Curry et al., 2005) [21]. Through KEGG enrichment analysis, differential metabolites were enriched into 44 metabolic pathways. And lipid metabolism is a key metabolic pathway in plant wax synthesis. In this experiment, differential metabolites were enriched in ether lipid metabolism, fatty acid biosynthesis, and α-The, the three metabolic pathways of linolenic acid metabolism.

A series of genes, *CsCER1*, *CsCER3*, *CsCER4*, *CsCER6*, *CsCER7*, *CsCER8*, *CsCER10*, *CSWAX2*, *CsWIN1*, *CsTT4*, *CsFLS1*, *CsCER26*, and *CsMYB36*, have been reported to be involved in the cucumber glossy phenotype. Liu et al. (2014) found that *CsCER7* responded to ABA and affected wax formation in the peel [11]. Wang (2015) compared *CER1* in Arabidopsis with the reference genome of cucumber to obtain the homologous gene *CsCER1*. It was found that *CsCER1* was located on the endoplasmic reticulum membrane and plays an important role in the biosynthesis of wax alkanes. *CsCER*1 was expressed in all tissues except the roots, and its expression in the epidermis was high. It was also found that different rootstock grafting methods affected the wax content of the cucumber scion fruit epidermis, and the expression of genes related to wax synthesis in the fruit epidermis also changed [22]. Four days after flowering, the expression levels of *CsCER1, CsCER3*, *CsCER6*, *CsCER8*, and *CsCER10* in black seed pumpkin-grafted plants were significantly higher than those in white seed pumpkin-grafted plants and self-rooted seedlings [23]. Wang et al. (2015) identified the gene *CsCER10* that may be related to wax synthesis screened by predecessors. Therefore, *CsCER10* is involved in wax synthesis. *CsCER4* may play an important role in the wax formation process. *CSWAX2* in cucumber plants has a significant impact on the paraffin content in wax powder through transgenic technology [24,25,26]. Some TFs that influence wax biosynthesis have also been identified in grafted cucumbers. Zhang et al. (2019) found the Apetala2/ethylene response factor (AP2/ERF)-type horizontal transcription factor CsWIN1, which further promotes the biosynthesis of several key waxes and the expression of transport proteins, resulting in a more shiny appearance of the gene [27]. In this study, we found that grafted cucumbers showed a glossy phenotype. The present study identified 68 DEGs in WG versus SR, 8 of which were annotated to the wax biosynthesis pathway, including *CER1*. Based on these results, we hypothesized that *CsCER1* plays an important role in cucumber biosynthesis. The potential wax transporter genes that are co-expressed with the key gene *CsCER1* were analyzed by the co-expression network. We hypothesized that *CsWRKY53* may be affected by grafting and could be a master switch that transcriptionally regulates the expression of wax biosynthesis pathway genes in response to grafting, specifically activating *CsCER1*. Other TFs, such as *CsTCP14*, may regulate the expression of wax biosynthesis pathway genes in response to grafting.

## 4. Materials and Methods

### 4.1. Plant Material

Both the rootstock variety “White Seed Pumpkin” and the cucumber scion variety “Zhongnong 18” were tested. There were two treatments in the experiment, that is, a self-rooted plant (SR) and white seed pumpkin-grafted cucumber (WG). The pumpkin seeds were sown on 19 August 2021. Cut grafting was performed when the cotyledons were flattened, and the true leaves were exposed. All the materials were planted in the greenhouse at Shenyang Agricultural University in September 2021. The row spacing of the plants was 35 × 60 cm, with 16 plants in each row. Other cultivation and management practices were carried out according to the conventional cucumber methods. On the 3rd, 6th, 9th, 12th, and 15th days after flowering, the cucumber fruits were picked, and the sampling node was the 10–14th node of the plant from the base of the grafted plant to the upper part of the ear cotyledon. The flowering date and nodes were marked in advance, and each treatment was repeated three times. A mixed sample of six cucumbers was collected each time; the fruit was uniform, free of pests and mechanical damage, and the days after flowering were the same. The fresh samples were selected to measure the brightness of the peel surface and wax powder. The samples used for RNA-seq and Quasi-Targeted Metabolomics were stored at −80 °C.

### 4.2. Surface Lightness Analysis and SEM Analysis

Relatively flat positions on the fruit surface were selected for surface lightness analysis using a color difference meter (CR-410). We measured brightness L1, and then the wax powder on the fruit surface was removed to measure brightness L2. The brightness difference ΔL was calculated using L1 minus L2. The upper, middle, and lower parts of each fruit surface were taken and repeated five times.

In the five sampling stages, 1 cm^2^ of cucumber peel with a thickness of 2–3 mm was sliced using a thin, sharp blade, and all the samples were instantly fixed with 2.5% glutaraldehyde. The samples were then washed three times for a total of 15 min with phosphoric acid buffer salt solution (PBS). They were then washed successively with gradient ethanol solutions for 20 min each. The samples were dried using a vacuum freeze drier and covered with a thin metal layer. The structure and morphology of the wax powder were observed using a scanning electron microscope (SEM, TM3030, Hitachi, Tokyo, Japan) at 200× magnification.

### 4.3. RNA-Seq Analysis

RNA was extracted from the fruit surfaces of WG and SR 12 d after flowering using the TRIzol method. RNA integrity was assessed using an RNA Nano 6000 Assay Kit on a Bioanalyzer 2100 system (Agilent Technologies, California, CA, USA). Six cDNA libraries were constructed and sequenced using the Illumina HiSeq platform (Illumina, San Diego, CA, USA) and 2 × 150 bp paired-end reads were generated.

After removing reads containing adapters, reads containing poly-N, and low-quality reads from raw data, the Q20, Q30, and GC contents of the clean data were calculated. These were then aligned to the reference genome using Hisat2 v2.0.5. The mapped reads for each sample were assembled using StringTie (v1.3.3b) with a reference-based approach [28]. The expected number of fragments per Kilobase (FPKM) of the transcript sequence per million base pairs sequenced considers the effect of sequencing depth and gene length on the read count simultaneously. It is currently the most used method for estimating gene expression levels. Differential expression analysis of the two groups was performed using the DESeq2 R package (version 1.20.0). Genes with an adjusted *p*-value ≤ 0.05 in DESeq2 were assigned as differentially expressed.

Gene Ontology (GO) enrichment analysis of differentially expressed genes was implemented using the cluster Profiler R package. GO terms with corrected *p*-values less than 0.05 were considered significantly enriched by differentially expressed genes. We also used the cluster Profiler R package to test the statistical enrichment of expression genes in the KEGG pathways.

### 4.4. Quasi-Targeted Metabolomics

Each tissue sample (100 mg) was ground with liquid nitrogen before being combined with pre-chilled 80% methanol using a vortex. After incubation on ice for 5 min, the samples were centrifuged at 15,000× *g* for 20 min. A portion of the supernatant was diluted using LC-MS chromatography-tandem mass spectrometry (LC-MS-grade water) to a final concentration of 53% methanol. The samples were then transferred to a brand-new Eppendorf tube and centrifuged there for 20 min at 15,000× *g*, 4 °C. The supernatant was then injected into the LC-MS/MS system.

LC-MS/MS analyses were performed using an ExionLC™ AD system (SCIEX) coupled with a QTRAP^®^ 6500+ mass spectrometer (SCIEX) in Novogene Co., Ltd. (Beijing, China). For the positive/negative polarity mode, the samples were injected onto an Xselect HSS T3 column (2.1150 mm, 2.5 m) using a 20 min linear gradient at a flow rate of 0.4 mL/min. The eluents used were eluents A (0.1% formic acid-water) and B (0.1% formic acid-acetonitrile). The solvent gradient was set as follows: 2% B, 2 min; 2–100% B, 15.0 min; 100% B, 17.0 min; 100–2% B, 17.1 min; 2% B, 20 min. QTRAP^®^ 6500+ mass spectrometer was operated in positive polarity mode with Curtain Gas of 35 psi, Collision Gas of Medium, IonSpray Voltage of 5500 V, Temperature of 550 °C, Ion Source Gas of 1:60, Ion Source Gas of 2:60. QTRAP^®^ 6500+ mass spectrometer was operated in negative polarity mode with Curtain Gas of 35 psi, Collision Gas of Medium, IonSpray Voltage of −4500 V, Temperature of 550 °C, Ion Source Gas of 1:60, Ion Source Gas of 2:60.

The KEGG (Kyoto Encyclopedia of Genes and Genomes) and LIPID MAPS databases were used to annotate these metabolites. We applied univariate analysis (*t*-test) to calculate the statistical significance (*p* value). The metabolites with VIP > 1 and *p*-value < 0.05 and fold change ≥ 2 or FC ≤ 0.5 were considered to be differential metabolites. Based on the Log_2_(FC) and log10 (*p*-value) of the metabolites using ggplot2 in the R language, volcano plots were used to filter the metabolites of interest. The cor function of the R language was used to assess the correlation between different metabolites (method = Pearson). Statistical significance was set at *p* < 0.05, and the R language corrplot tool was used to generate correlation plots.

### 4.5. Correlation Analysis of Differential Genes and Differential Metabolites

Correlation analysis of differential genes and differential metabolites was based on the Pearson statistical method to calculate the correlation coefficients r^2^ and *p* value of differential genes and differential metabolites. The correlation analysis was conducted between the differential metabolites displayed Top50 (*p*-value sorted from small to large) and the differential genes displayed Top50 (*p*-value sorted from small to large). The differential genes and metabolites were mapped to the KEGG pathway database, their common path information was obtained, and the main biochemical and signal transduction pathways in which the differential metabolites and differential genes participated were identified.

### 4.6. qRT-PCR Analysis

The relative expression levels of 24 candidate genes were measured using gene-specific primers and real-time quantitative PCR (qRT-PCR). All the gene-specific primers were designed using Primer 5.0 (Table S3). The same RNA samples used for RNA-seq were also used for qRT-PCR. Each RNA sample was analyzed in triplicate. qRT-PCR was performed using a *PerfectStart* Uni RT & qPCR Kit (TransGen Biotech, Dalian, China) and a LightCycle 96 PCR system (Roche, Switzerland). The 20 µL reactions contained 2 µL of cDNA, 0.4 µL of each pair of target primers (200 nM), 7.2 µL of Nuclease-Free Water, and 10 µL of 2× PerfectStart Green qPCR SuperRealMix (TransGen Biotech, Suzhou, China). PCR conditions were as follows: 95 °C for 15 min (preincubation); 40 cycles of 95 °C for 10 s and 60 °C for 30 s (2-step amplification); 95 °C for 10 s, 65 °C for 60 s, and 95 °C for 1 s (melting). Relative gene expression levels were analyzed according to the 2^−∆∆Ct^ method. The internal normalization gene used was *CsUbquitin*.

## 5. Conclusions

**Figure 10:**
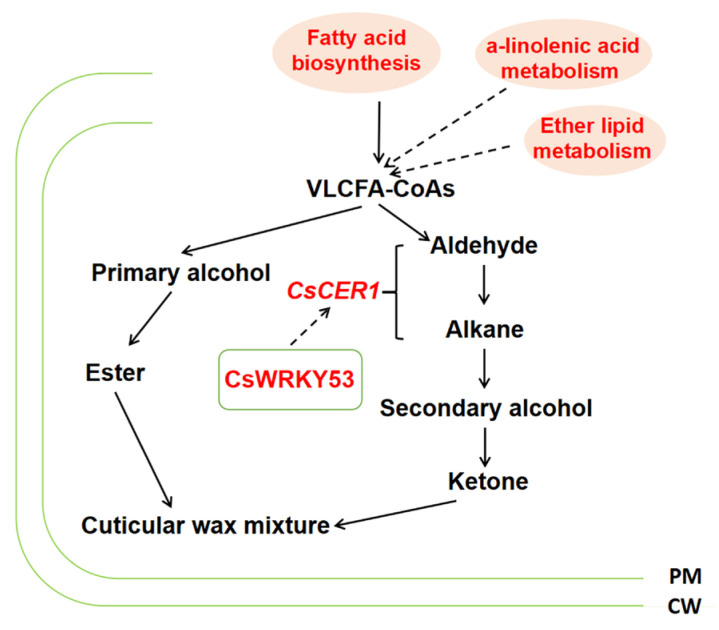
The mechanism of the glossy phenotype formation. The solid arrow represents the determined upstream and downstream relationships, while the required arrow represents the inferred interaction relationships.

In conclusion, a significant wax deficiency was observed in cucumbers in response to grafting. Morphological and physiological comparisons indicated that the rootstock affected the wax biosynthesis in the scions. Using transcriptomic and LC-MS analyses, we identified several differentially expressed structural genes and transcription factors that are potentially involved in wax biosynthesis. We hypothesized that *CsWRKY53* may be affected by grafting as a master switch that transcriptionally regulates the expression of wax biosynthesis pathway genes in response to grafting, specifically by activating *CsCER1*. We also found that fatty acid biosynthesis, ether lipid metabolism, and a-linolenic acid metabolism may also affect the wax biosynthesis in cucumber in response to grafting (Figure 10). These results have provided a theoretical basis for screening wax biosynthesis genes in cucumber in response to grafting. This study provides data highlighting the regulatory mechanisms of wax biosynthesis genes in cucumbers in response to grafting.

## Figures and Tables

**Table 1:** Mean value of brightness difference between WG and SR from different sampling periods.

Treatment	3 d	6 d	9 d	12 d	15 d
SR	1.03 ± 0.02	1.12 ± 0.02	1.24 ± 0.01	1.35 ± 0.12	1.23 ± 0.06
WG	1.09 ± 0.03	1.14 ± 0.02	1.07 ± 0.15 **	0.98 ± 0.03 **	0.96 ± 0.08 **

** indicates *p* < 0.01.

**Table 2:** Expression results of differentially related genes involved in wax synthesis.

Gene Category	Gene ID	Gene Description	WG Average Count	SR Average Count	log2Fold Change
Wax biosynthesis	*CsaV3_3G010290*	Protein ECERIFERUM 1-like	131.00	0.67	7.52
	*CsaV3_3G010300*	Protein ECERIFERUM 1-like	122.67	39.33	1.55
	*CsaV3_6G014920*	Fatty acyl-CoA reductase	157.67	0.00	9.65
	*CsaV3_6G014930*	Fatty acyl-CoA reductase	1136.67	411.33	1.37
Cutin and suberin biosynthesis	*CsaV3_6G049160*	Cytochrome P450 77A4	3.67	16.33	−2.25
	*CsaV3_6G000610*	Cytochrome P450 86A2	355.33	740.33	−1.15
	*CsaV3_3G043880*	GMC oxidoreductase	947.00	2027.67	−1.19
	*CsaV3_1G000360*	Omega-hydroxypalmitate O-feruloyl transferase	21.33	3.67	2.47
Diterpenoid biosynthesis	*CsaV3_3G049410*	Cytochrome P450 88D6	579.00	146.00	1.89
	*CsaV3_3G049400*	Cytochrome P450 88D6	773.00	262.33	1.45
	*CsaV3_3G049420*	Cytochrome P450 88D6	102.67	14.33	2.73
	*CsaV3_3G010170*	Cytochrome P450 88A3	20.67	6.00	1.70
Sesquiterpenoid and triterpenoid biosynthesis	*CsaV3_3G005740*	Alpha-farnesene synthase	330.67	27.33	3.50
	*CsaV3_3G003680*	(−)-germacrene D synthase	158.00	18.33	3.01
	*CsaV3_1G011420*	(3S,6E)-nerolidol synthase 1-like	64.67	6.00	3.32
	*CsaV3_3G005780*	(+)-gamma-cadinene synthase	131.67	54.67	1.15
Biosynthesis of unsaturated fatty acids	*CsaV3_3G038870*	Delta(12)-fatty-acid desaturase FAD2	5973.33	11,245.67	−1.00
	*CsaV3_6G030990*	Stearoyl-[acyl-carrier-protein] 9-desaturase 6	281.33	55.00	2.27
	*CsaV3_UNG137080*	Acyl-coenzyme A oxidase	31.33	8.00	1.89
Fatty acid degradation	*CsaV3_7G024990*	Alcohol dehydrogenase class-P	2866.67	329.00	3.03
	*CsaV3_1G001600*	Aldehyde dehydrogenase family 2 member B4	5782.00	2553.33	1.09
	*CsaV3_7G024950*	Alcohol dehydrogenase 1	412.33	151.33	1.36
	*CsaV3_4G005260*	Aldehyde dehydrogenase family 3 member F1	177.67	66.33	1.31
Steroid biosynthesis	*CsaV3_3G046140*	24-methylenesterol C-methyltransferase 2	152.33	413.33	−1.53
	*CsaV3_3G027160*	Triacylglycerol lipase 2	69.00	14.67	2.14
	*CsaV3_4G028790*	Delta(7)-sterol-C5(6)-desaturase	32.00	8.33	1.84
Alpha-linolenic acid metabolism	*CsaV3_5G035840*	4-coumarate-CoA ligase-like 5	446.33	196.67	1.09
	*CsaV3_4G023820*	Linoleate 13S-lipoxygenase 2-1	125.00	282.33	−1.27
Glycerophospholipid metabolism	*CsaV3_3G026730*	Glycerophosphodiester phosphodiesterase GDPD1	3142.33	694.00	2.09
	*CsaV3_1G007140*	Phosphoethanolamine N-methyltransferase 1	2040.33	648.67	1.57
	*CsaV3_3G032790*	Phosphatidyl-N-methylethanolamine N-methyltransferase	1937.33	882.00	1.04
	*CsaV3_4G002900*	Glycerophosphodiester phosphodiesterase GDPD3	466.00	182.67	1.26
	*CsaV3_1G043430*	Non-specific phospholipase C2	277.33	94.33	1.47
	*CsaV3_5G033710*	Choline/ethanolaminephosphotransferase 1	347.33	140.67	1.22
	*CsaV3_5G034660*	Lipid phosphate phosphatase 2	62.33	28.33	1.04
Glycerolipid metabolism	*CsaV3_3G015560*	Diacylglycerol acyltransferase	270.00	678.33	−1.42
	*CsaV3_7G032960*	D-glycerate 3-kinase	19.33	48.00	−1.42
Linoleic acid metabolism	*CsaV3_2G006460*	Linoleate 9S-lipoxygenase 6	2531.00	106.67	4.48
Arachidonic acid metabolism	*CsaV3_6G045790*	Gamma-glutamyltranspeptidase 1	120.00	49.67	1.18
Sphingolipid metabolism	*CsaV3_6G016520*	Neutral ceramidase	611.00	238.33	1.26
Fatty acid elongation	*CsaV3_1G033910*	3-ketoacyl-CoA synthase 12	417.33	169.33	1.20

## Data Availability

The raw data from the transcriptome analysis were deposited in the SRA database under accession number PRJNA987737 (accessed on 3 July 2023).

## Supplementary Materials

The following supporting information can be downloaded at: https://www.mdpi.com/article/10.3390/ijms241512147/s1.
