# Supplementary material for: Integrated Metabolome and Transcriptome Analysis Provides New Insights into the Glossy Graft Cucumber Fruit (Cucumis sativus L.)
Source: Int J Mol Sci. 2023 Jul 29;24(15):12147. doi: 10.3390/ijms241512147 (PMC10418779; doi:10.3390/ijms241512147)
Supplement: Supplementary file 1 [file ijms-24-12147-s001.zip › ijms-2502529-supplementary.pdf]

Table S1. Summary of sample sequencing data quality.

sample	raw_reads	raw_bases	clean_reads	clean_bases	error_rate	Q20	Q30	GC_pct
WG12A	46706414	7.01G	43189142	6.48G	0.03	97.39	92.99	44.64
WG12B	44810140	6.72G	41568272	6.24G	0.03	97.63	93.37	44.9
WG12C	45670848	6.85G	42737406	6.41G	0.03	97.05	92.14	44.2
SR12A	43047992	6.46G	39284802	5.89G	0.03	97.34	92.83	44.19
SR12B	43439006	6.52G	42038676	6.31G	0.03	96.83	91.7	44.99
SR12C	45909026	6.89G	44501570	6.68G	0.03	96.99	92.04	45.08

Table S2. Comparison statistics of samples and reference genome.

sample	total_reads	total_map	unique_map	multi_map	read1_map	read2_map	positive_map	negative_map	splice_map	unsplice_map	proper_map
WG12A	43189142	40941746 (94.8%)	39480226 (91.41%)	1461520 (3.38%)	19827224 (45.91%)	19653002 (45.5%)	19711924 (45.64%)	19768302 (45.77%)	15825473 (36.64%)	23654753 (54.77%)	37008410 (85.69%)
WG12B	41568272	39842628 (95.85%)	38470016 (92.55%)	1372612 (3.3%)	19292620 (46.41%)	19177396 (46.13%)	19216876 (46.23%)	19253140 (46.32%)	15222650 (36.62%)	23247366 (55.93%)	36540914 (87.91%)
WG12C	42737406	40433707 (94.61%)	39080059 (91.44%)	1353648 (3.17%)	19671254 (46.03%)	19408805 (45.41%)	19512854 (45.66%)	19567205 (45.78%)	15799181 (36.97%)	23280878 (54.47%)	36324608 (84.99%)
SR12A	39284802	37365944 (95.12%)	36186668 (92.11%)	1179276 (3.0%)	18182532 (46.28%)	18004136 (45.83%)	18069260 (46.0%)	18117408 (46.12%)	14245020 (36.26%)	21941648 (55.85%)	33269454 (84.69%)
SR12B	42038676	40256123 (95.76%)	39000235 (92.77%)	1255888 (2.99%)	19668763 (46.79%)	19331472 (45.98%)	19484024 (46.35%)	19516211 (46.42%)	15020324 (35.73%)	23979911 (57.04%)	37443206 (89.07%)
SR12C	44501570	42589888 (95.7%)	41226579 (92.64%)	1363309 (3.06%)	20750728 (46.63%)	20475851 (46.01%)	20595242 (46.28%)	20631337 (46.36%)	16005154 (35.97%)	25221425 (56.68%)	39694584 (89.2%)

Table S3. Comparison statistics of samples and reference genome.

Gene ID	Forward primer 5'-3'	Reverse primer 3'-5'
CsaV3_1G001250	AGAGCATGTAGTGGCACTTTGA	AGAGCATGTAGTGGCACTTTGA
CsaV3_1G011420	TAGATGGGACTTGGCTGCTG	TGCCTCAACCAGAAATGCCT
CsaV3_1G000360	TTTGTCATATGGGGTGGGGC	TGGAATCCTAGCCTCGACCA
CsaV3_1G010980	TGCTTGGGCAAATTGGAAGC	TATAGCGGCGTTTCTCGTCC
CsaV3_1G022900	TCTACGCAGGGAGTCATGGA	CTTGCCGGTTGGAAAAGCAC
CsaV3_1G041400	AGCAGGCCTCAAGCGTATTT	GCGGTCGAGGTTGGATACAT
CsaV3_2G001890	TTCTCAGATTTGCCACCGCT	TGTGAGTGGAGATGCGTGAC
CsaV3_2G007750	GAGGAGAAGACTCATCGGCG	TGCACCTTCGATTCCGTTCA
CsaV3_2G012080	GTTGGTTGGGCGGAAAACTC	TTCCCATTTTCCGGCACGTA
CsaV3_3G003680	AGATAGGGCTGTGGAGTGCTA	CTTCGAAGGTGGCATAGGCA
CsaV3_3G005740	AGCGACAAAGGAGCTTGGAA	CCATGTGTGAACCCATCCCT
CsaV3_3G010290	ACCACTACACCCCTGCATTG	GCTCTGTCCACCCTTCCATC

CsaV3_3G010300	CTCGGATCGTAGACAAGGGC	TCCGACCTTTCGCCGTTAAA
CsaV3_3G043270	TCTTGCAGCCTACCCTGTTG	AGCCGTGGTCGAAAGATCAG
CsaV3_3G049410	AGGGTCTCAACTTGATGCCAA	GGCGACAACCAATTTGTCCA
CsaV3_3G049420	TCATGCACTCAAGGAAGAGCA	TGCTGTTGCCTCACGAAAAC
CsaV3_4G028640	GACAGATCGCGGACAAATGC	CTGGGCCCTCGAAGACTTTT
CsaV3_4G037260	TACACAGGAAAGCGCGAGAG	CATCCACCCATCTACCCACG
CsaV3_6G000890	GCAATGCGGGGCTTTTTGTA	ACACGAAAGGATGAGCCAGG
CsaV3_6G014920	TCGGTTACGATATGGTGGTGA	AGAAAGCCCGTCAAATCCCC
CsaV3_6G030990	GGCTAATGACTACGCGGACA	CCCATGGGGCTTCAACTTCT
CsaV3_6G045560	CCCCAATTGTCGATGACCCA	CTTGTCTCTACGTCTCCCGC
CsaV3_6G049160	GACAGGTTCTTGGAAGGCGA	CAACGTGCCCAAAGTCATGG
CsaV3_7G024990	AAGGGTCAATCCGTTGCGAT	CTTCTTCGAAACGAGCCGGA
Ubquitin (*CsaV3_3G0377000*)	TTTATATGCGTTCGTGGACTGG	CTTGGTGGCTTCTCAGGGTAAT
